# Supplementary material for: Drosophila TNFRs Grindelwald and Wengen bind Eiger with different affinities and promote distinct cellular functions
Source: Nat Commun. 2021 Apr 6;12:2070. doi: 10.1038/s41467-021-22080-9 (PMC8024323; doi:10.1038/s41467-021-22080-9)
Supplement: Supplementary file 1 — Supplementary Information [file 41467_2021_22080_MOESM1_ESM.pdf]

Drosophila TNFRs Grindelwald and Wengen bind Eiger with different affinities and promote distinct cellular functions

Palmerini et al.

SUPPLEMENTARY INFORMATION

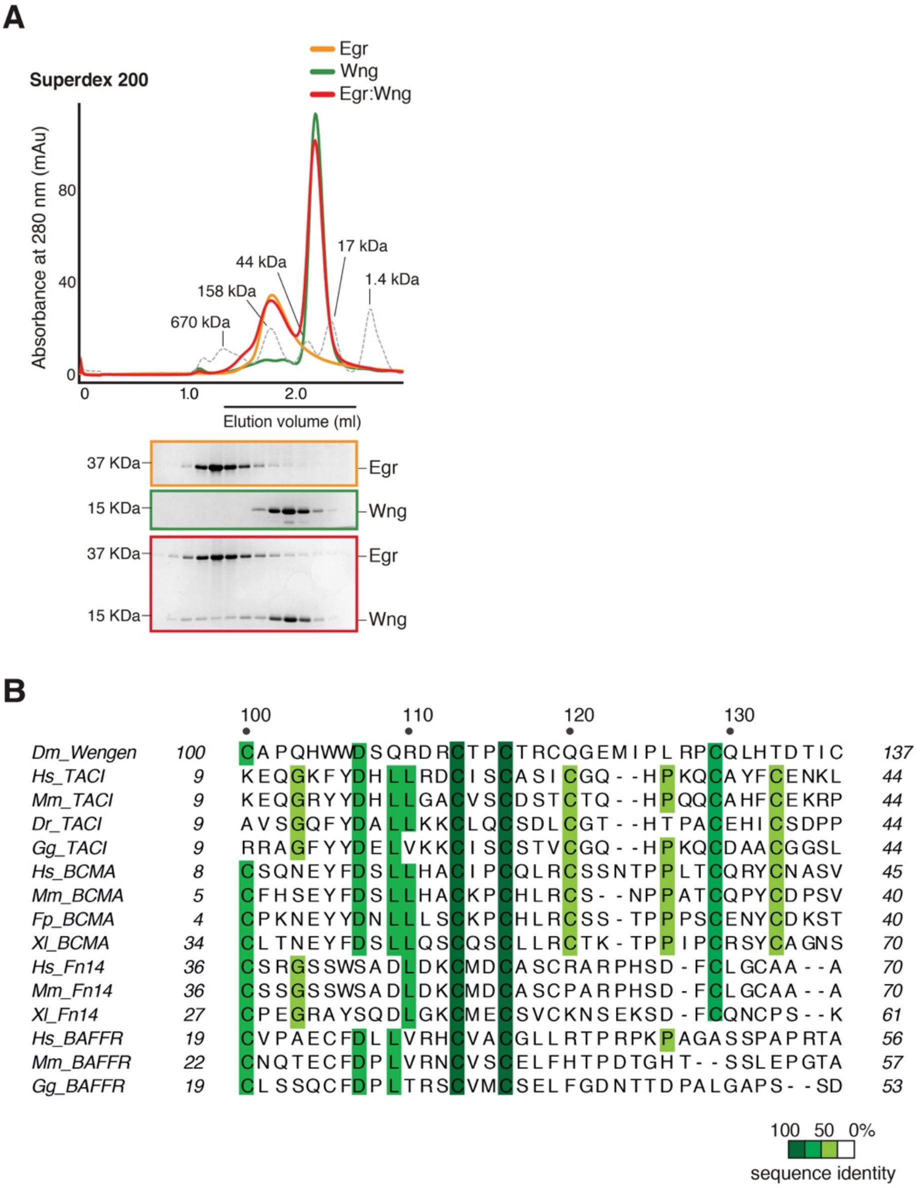

Supplementary Figure S1. Characterization of the Wng extra-cellular domain. A Analytical SEC of the Egr$^{146-409}$:Wng$^{78-201}$ complex at 30 µM concentration. Peak fractions were separated on SDS-PAGE and Coomassie-stained. Results of SEC runs and SDS-PAGES were confirmed by successful replicate experiments. **B** Sequence alignment of the CRD of

Wng with human Fn14, BCMA, BAFFR, and the second CRD of TACI and their orthologues, coloured by percentage of sequence identity.

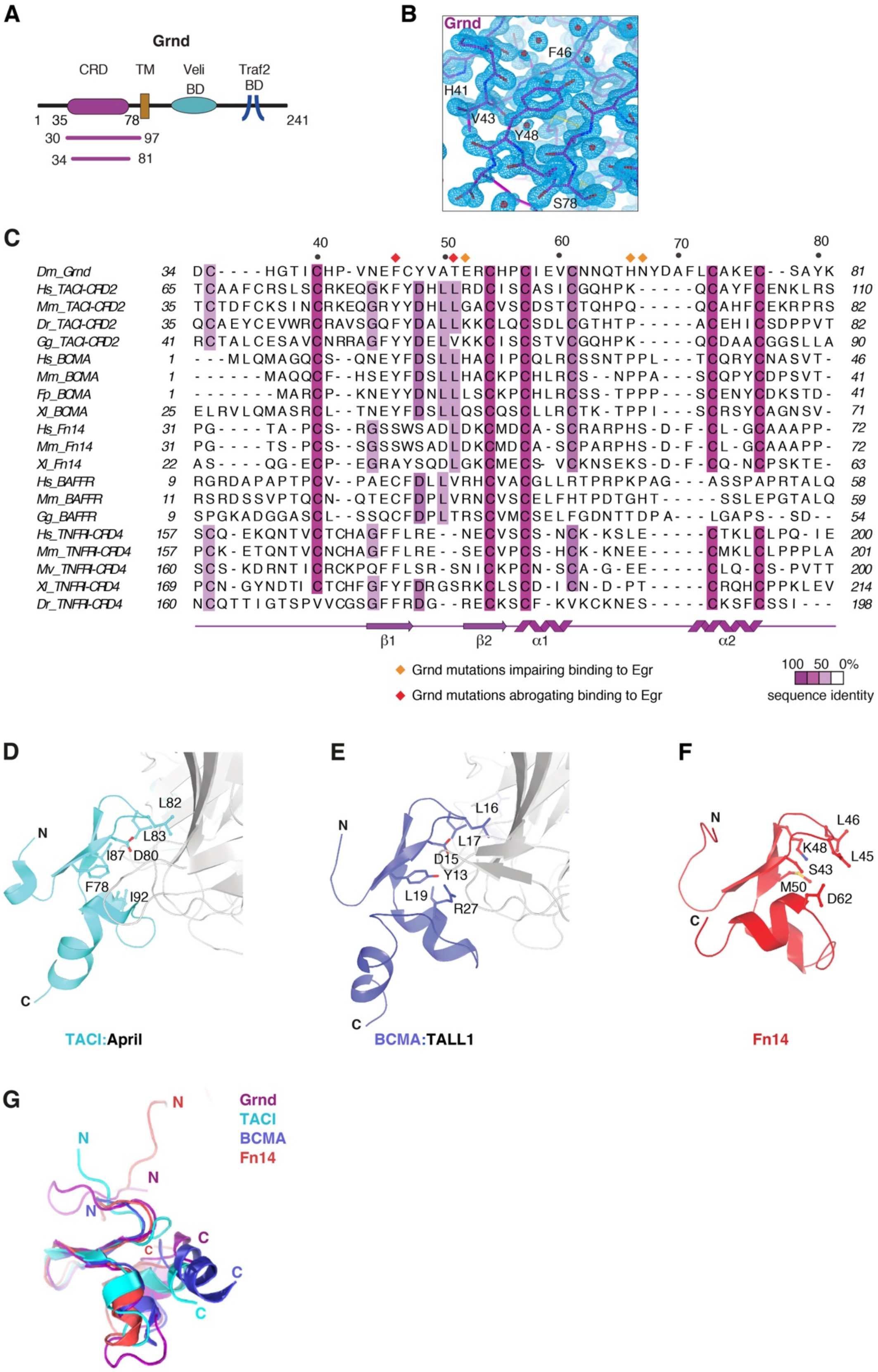

Supplementary Figure S2. Organization of the Grnd extra-cellular domain. A Domain structure of Grnd, showing the construct designed initially on the basis of primary sequence analysis, and the one obtained by limited proteolysis and used for structure determination. **B** Representative view of 2Fo-Fc electron density map contoured at 1.5 σ level for Grnd-ECD centred around Phe-46 (balls-and-stick representation in purple). Water solvent molecules are depicted as red spheres. **C** Sequence alignment of the CRD of Grnd with orthologues of Fn14, BCMA, BAFFR, the second CRD of TACI, and the forth CRD of TNFRI, coloured by percentage of sequence identity. The secondary structure elements of Grnd-ECD derived from the crystallographic structure are displayed below the sequences. **D-E** Cartoon representation of complexes between the human TNF ligands and single CRD-receptors folding as Grnd, visualized with the same orientation of Fig. 2B. In D, April:TACI-CRD2 (PDB ID 1XU1, ligand in grey, receptor in cyan); in E, TALL1:BCMA (PDB ID 10QD, ligand in grey, receptor in blue). **F** Structure of Fn14-ECD (PDB ID 2RPJ, in red). For all receptors, residues involved in ligand binding are shown in balls-and-sticks. **G** Cartoon representation of the superposition of Grnd-ECD (purple) with TACI (cyan), BCMA (blue) and Fn14 (red) based on the largest rigid body common to all structures calculated by the Rapido server (http://rapido.embl-hamburg.de/). The structural alignment carried out this way showed that Grnd/TACI superpose with and RMSD of 5.2 Å, while Grnd/BCMA and Grnd/Fn14 superpose with and RMSD of 4.4 Å.

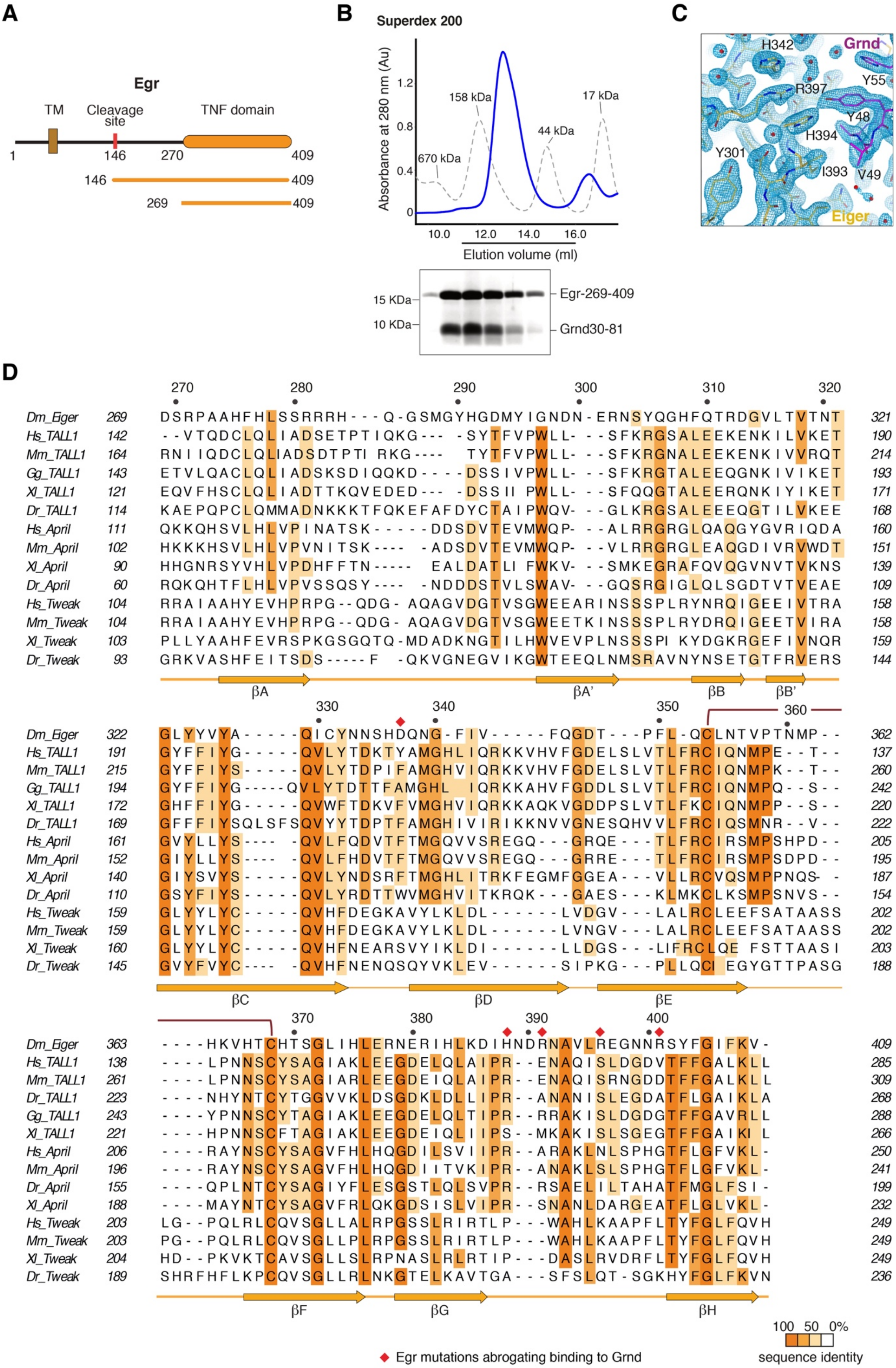
A
Egr
TM
Cleavage site
TNF domain
1
146
270
409
146
409
269
409
B
Superdex 200
Absorbance at 280 nm (Au)
1.2
0.8
0.4
0
158 kDa
17 kDa
44 kDa
670 kDa
10.0
12.0
14.0
16.0
Elution volume (ml)
15 KDa
10 KDa
Egr-269-409
Grnd30-81
C
H342
Grnd
Y55
R397
Y48
H394
Y301
I393
V49
Eiger
D
Dm_Eiger
Hs_TALL1
Mm_TALL1
Gg_TALL1
Xl_TALL1
Dr_TALL1
Hs_April
Mm_April
Xl_April
Dr_April
Hs_Tweak
Mm_Tweak
Xl_Tweak
Dr_Tweak
βA
βA'
βB
βB'
βC
βD
βE
βF
βG
βH
100 50 0%
sequence identity
Egr mutations abrogating binding to Grnd

Supplementary Figure S3. Sequence analysis of the Egr TNF-homology domain. A Domain structure of Egr, showing the constructs encompassing the entire extra-cellular portion after TACE cleavage, or obtained by limited proteolysis for structure determination. **B** Analytical SEC of the trypsinized Eiger$^{269\text{-}409}$:Grnd$^{30\text{-}81}$ complex showing that the trimmed constructs still form hetero-hexamers. Peak fractions were separated on SDS-PAGE and Coomassie-stained. The SEC run and SDS-PAGE were confirmed by successful replicate experiments. **C** Representative view of 2Fo-Fc electron density map at the Egr:Grnd interface contoured at 1.5 σ level (blue) with balls-and-sticks representation of the corresponding Eiger (yellow) and Grnd (purple) chains. Water solvent molecules are depicted as red spheres. **D** Sequence alignment of the Eiger TNF-homology domain with amino acids coloured by sequence identity calculated on *Drosophila melanogaster* Egr (short isoform) and orthologues of TALL1, April and Tweak from *Homo sapiens*, *Mus musculus*, *Gallus gallus*, *Danio rerio*, and *Xenopus laevis*. Secondary structure elements of Egr are displayed below the sequences. Residues essential for Grnd binding are marked by red rhombuses.

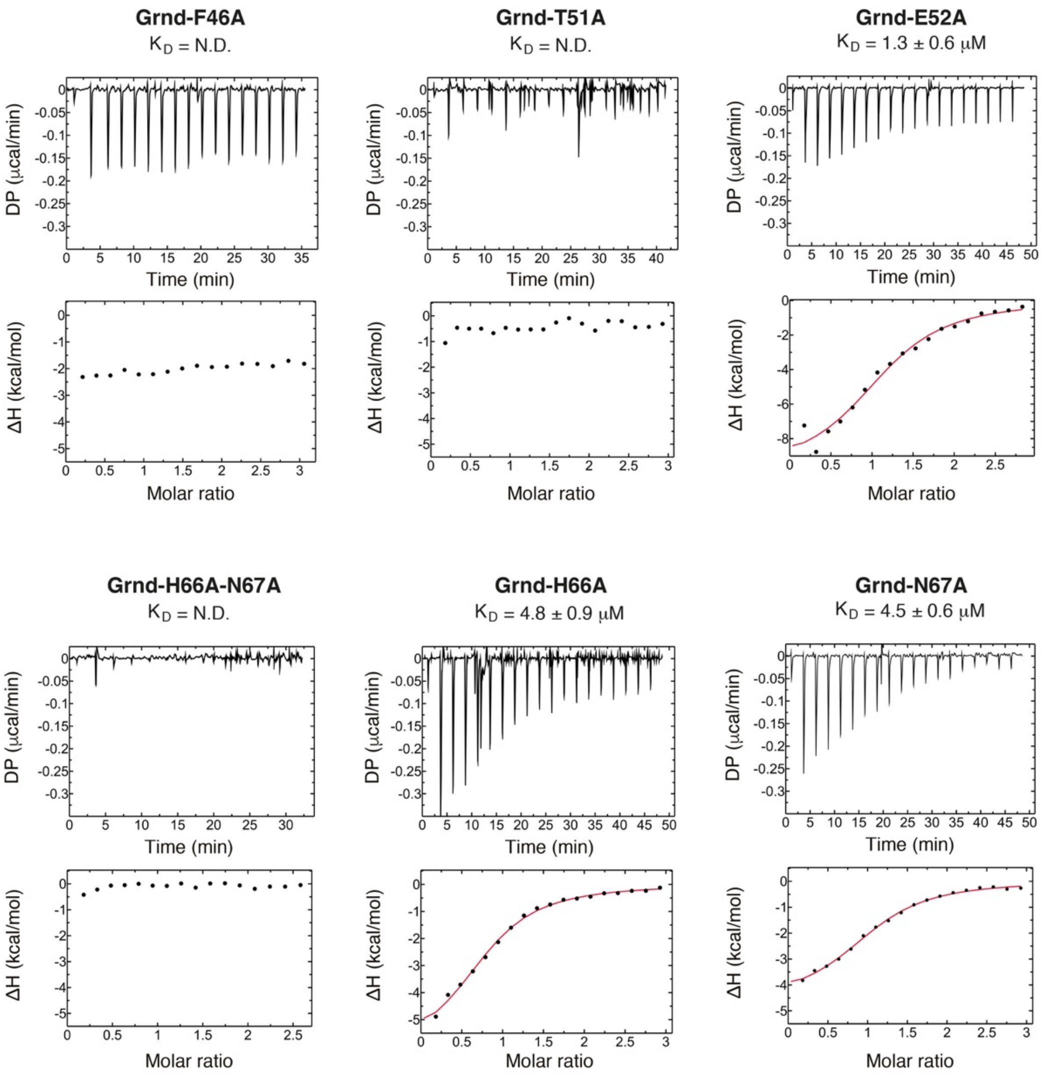

Supplementary Figure S4. Thermodynamic characterization of the Eiger:Grnd interaction. ITC measurements of the binding affinity between $Egr^{146-409}$ and $Grnd^{30-97}$ carrying the indicated mutations. K_D is reported as mean ± error fitting of the ITC data with the isotherm (red line). All reactions are exothermic.

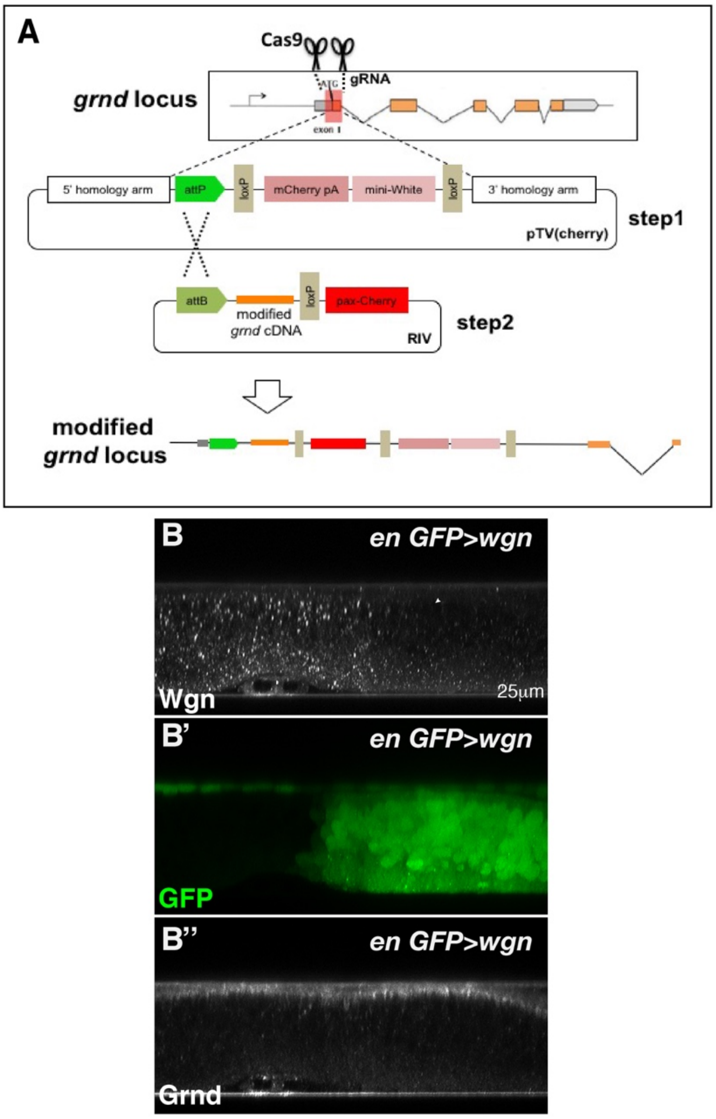

Supplementary Figure S5. Egr and Wgn localise to distinct vesicles. A *grnd* editing by accelerated homologous recombination. Schematics illustrating the different steps employed to edit the *grnd* locus. The editing allows replacing the first exon of *grnd* (the area between the two scissors) with cDNAs encoding wildtype *grnd* or *grnd* harbouring different point mutations. For detailed description see Methods. **B-B''** Transversal sections of dissected wing discs driving the expression of Wgn RNAi in the engrailed domain (labelled in green), showing that the majority of Wgn localises to intracellular vesicles. Wgn knockdown does not change the localisation of Grnd (B''). Results presented in panel B-B" were confirmed by successful replicate experiments.

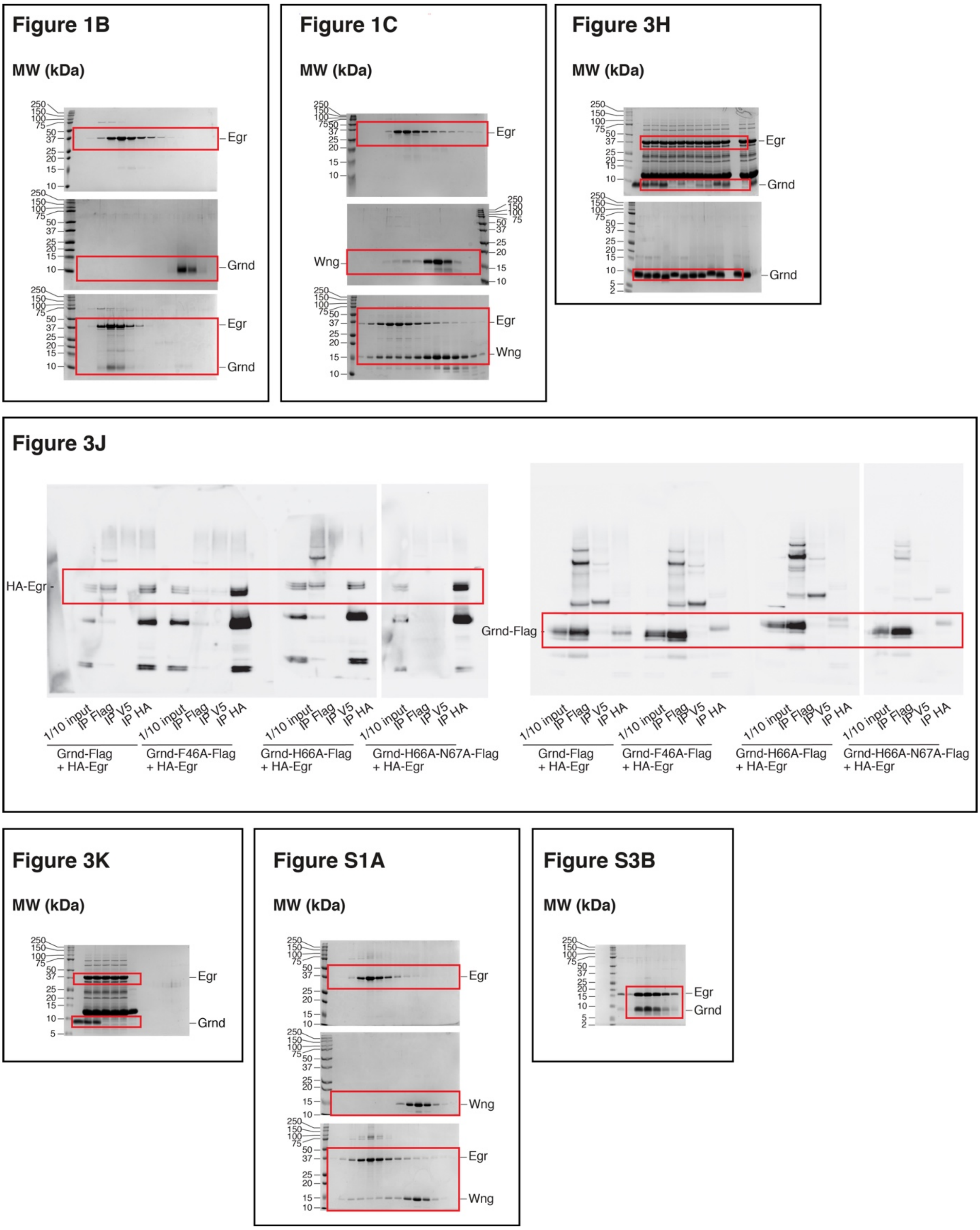

Supplementary Figure S6. Uncropped images of SDS-PAGES and immunoblots presented in the main and supplementary figures.

PRIMER NAME	SEQUENCE	VECTOR
**BIOCHEMISTRY**		
Grnd30_For	CGCGCGCCATGGGTGGAGAGAGTAGGGATTGCCAT	pETM14-His-Grnd30-97
Grnd97_Rev	GCGCGCGTCGACTTACTGGGTATTCTGGATGTCCAG	pETM14-His-Grnd30-97
Grnd-H36A_For	GAGAGAGTAGGGATTGCGCTGGCACGATCTGTCATG	pETM14-His-Grnd30-97-H36A
Grnd-H36A_Rev	CATGACAGATCGTGCCAGCGCAATCCCTACTCTCTC	pETM14-His-Grnd30-97-H36A
Grnd-F46A_For	CTGTCATCCGGTCAATGAAGCATGCTATGTTGCAACGGAGAG	pETM14-His-Grnd30-97-F46A
Grnd-F46A_Rev	CTCTCCGTTGCAACATAGCATGCTTCATTGACCGGATGACAG	pETM14-His-Grnd30-97-F46A
Grnd-Y48A_For	CCGGTCAATGAATTTTGCGCTGTTGCAACGGAGAGATGT	pETM14-His-Grnd30-97-Y48A
Grnd-Y48A_Rev	ACATCTCTCCGTTGCAACAGCGCAAAATTCATTGACCGG	pETM14-His-Grnd30-97-Y48A
Grnd-T51A_For	GAATTTTGCTATGTTGCAGCGGAGAGATGTCATCCGTG	pETM14-His-Grnd30-97-T51A
Grnd-T51A_Rev	CACGGATGACATCTCTCCGCTGCAACATAGCAAAATTC	pETM14-His-Grnd30-97-T51A
Grnd-E52A_For	TTTGCTATGTTGCAACGGCGAGATGTCATCCGTGCAT	pETM14-His-Grnd30-97-E52A
Grnd-E52A_Rev	ATGCACGGATGACATCTCGCCGTTGCAACATAGCAAA	pETM14-His-Grnd30-97-E52A
Grnd-H66A-N67A_For	TCTGCAATAATCAAACCGCCGCCTACGATGCGTTTCTGTGCGC	pETM14-His-Grnd30-97-H66A-N67A
Grnd-H66A-N67A_Rev	GCGCACAGAAACGCATCGTAGGCGGCGGTTTGATTATTGCAGA	pETM14-His-Grnd30-97-H66A-N67A
Grnd-H66A_For	GGTCTGCAATAATCAAACCGCCAACTACGATGCGTTTCTG	pETM14-His-Grnd30-97-H66A
Grnd-H66A_Rev	CAGAAACGCATCGTAGTTGGCGGTTTGATTATTGCAGACC	pETM14-His-Grnd30-97-H66A
Grnd-N67A_For	GCAATAATCAAACCCACGCCTACGATGCGTTTCTGTGC	pETM14-His-Grnd30-97-N67A
Grnd-N67A_Rev	GCACAGAAACGCATCGTAGGCGTGGGTTTGATTATTGC	pETM14-His-Grnd30-97-N67A
Grnd-F71A_For	ACCCACAACTACGATGCGGCTCTGTGCGCCAAGGAATGT	pETM14-His-Grnd30-97-F71A
Grnd-F71A_Rev	ACATTCCTTGGCGCACAGAGCCGCATCGTAGTTGTGGGT	pETM14-His-Grnd30-97-F71A
Grnd-K75A_For	GATGCGTTTCTGTGCGCCGCGGAATGTTCAGCTTACAA	pETM14-His-Grnd30-97-K75A
Grnd-K75A_Rev	TTGTAAGCTGAACATTCCGCGGCGCACAGAAACGCATC	pETM14-His-Grnd30-97-K75A
Eiger-146_For	CGCGCGAGATCTGGCGCAGACTACGAGGAC	pET43-His-Eiger146-409
Eiger-409_Rev	CGCGCGGTCGACTTACACCTTGAAGATGCCAAAGTA	pET43-His-Eiger146-409
Eiger-409_Rev	CGCGCGGTCGACTTACACCTTGAAGATGCCAAAGTA	pET100-Strep-Eiger146-409
Eiger-146_For	CGCGGCTAGCGGCGCAGACTACGAGGAC	pET100-Strep-Eiger146-409
Eiger-N333A_For	GCCCAGATATGCTACGCCAACTCGCACGACCAG	pET100-Strep-Eiger146-409-N333A
Eiger-N333A_Rev	CTGGTCGTGCGAGTTGGCGTAGCATATCTGGGC	pET100-Strep-Eiger146-409-N333A
Eiger-D337A_For	CTACAACAACTCGCACGCCCAGAACGGATTTATCG	pET100-Strep-Eiger146-409-D337A
Eiger-D337A_Rev	CGATAAATCCGTTCTGGGCGTGCGAGTTGTTGTAG	pET100-Strep-Eiger146-409-D337A
Eiger-H338A-R391A_For	ATCCATCTGAAGGACATTGCCAACGATGCCAATGCAGTTCTGCGG	pET100-Strep-Eiger146-409-H338A-R391A
Eiger-H338A-R391A_For	CCGCAGAACTGCATTGGCATCGTTGGCAATGTCCTTCAGATGGAT	pET100-Strep-Eiger146-409-H338A-R391A
Eiger-R396A_For	TCGCAATGCAGTTCTGGCGGAGGGAAACAACCGAA	pET100-Strep-Eiger146-409-H396A-R401A
Eiger-R396A_Rev	TTCGGTTGTTTCCCTCCGCCAGAACTGCATTGCGA	pET100-Strep-Eiger146-409-H396A-R401A
Eiger-R401A_For	TGGCGGAGGGAAACAACGCAAGCTACTTTGGCATCTT	pET100-Strep-Eiger146-409-H396A-R401A
Eiger-R401A_Rev	AAGATGCCAAAGTAGCTTGCGTTGTTTCCCTCCGCCA	pET100-Strep-Eiger146-409-H396A-R401A
Wng-78_For	CGCGCGCCATGGGTAACACAGACATAGCGCCA	pETM14-His-Wng78-201
Wng-201_Rev	CGCGCGCTCGAGTCAGCCAGTCTGCCAGTCAAG	pETM14-His-Wng78-201
**FLIES**		
grnd-5′-KO-pCFD4-For	TATATAGGAAAGATATCCGGGTGAACTTCgagcgtctgggccgcgttacGTTTTAGAGCTAGAAATAGCAAG	pCFD4
grnd-3′-KO-pCFD4-Rev	ATT TTAACTTGCTAT TTCTAGCTCTAAAACtgaacttgcatagaacccgccGACGTTAAATTGAAAATAGGTC	pCFD4
Homology-arm-I-sense	GCGGCCGCCGTATAGTTCATATTGGGATACTGGGAATTTC	
Homology-arm-I-anti	GGTACCGCGGCCCAGACGCTAAC	
Homology-arm-II-sense	ACTAGTAGGTTTTTCCAGCTGGACTTAATTG	
Homology-arm-II-anti	GGCGCGCCCATAACCGTTGTGGGCGTGG	

Supplementary Table S1. PCR primers.
