# Supplementary material for: Drosophila TNFRs Grindelwald and Wengen bind Eiger with different affinities and promote distinct cellular functions
Source: Nat Commun. 2021 Apr 6;12:2070. doi: 10.1038/s41467-021-22080-9 (PMC8024323; doi:10.1038/s41467-021-22080-9)
Supplement: Supplementary file 3 — Reporting Summary [file 41467_2021_22080_MOESM3_ESM.pdf]

nature research

Corresponding author(s): Marina Mapelli

Last updated by author(s): Feb 18, 2021

Reporting Summary

Nature Research wishes to improve the reproducibility of the work that we publish. This form provides structure for consistency and transparency in reporting. For further information on Nature Research policies, see our Editorial Policies and the Editorial Policy Checklist.

Statistics

For all statistical analyses, confirm that the following items are present in the figure legend, table legend, main text, or Methods section.

n/a	Confirmed	
☐	☒	The exact sample size (*n*) for each experimental group/condition, given as a discrete number and unit of measurement
☒	☐	A statement on whether measurements were taken from distinct samples or whether the same sample was measured repeatedly
☒	☐	The statistical test(s) used AND whether they are one- or two-sided<br>*Only common tests should be described solely by name; describe more complex techniques in the Methods section.*
☒	☐	A description of all covariates tested
☒	☐	A description of any assumptions or corrections, such as tests of normality and adjustment for multiple comparisons
☐	☒	A full description of the statistical parameters including central tendency (e.g. means) or other basic estimates (e.g. regression coefficient) AND variation (e.g. standard deviation) or associated estimates of uncertainty (e.g. confidence intervals)
☒	☐	For null hypothesis testing, the test statistic (e.g. *F*, *t*, *r*) with confidence intervals, effect sizes, degrees of freedom and *P* value noted<br>*Give P values as exact values whenever suitable.*
☒	☐	For Bayesian analysis, information on the choice of priors and Markov chain Monte Carlo settings
☒	☐	For hierarchical and complex designs, identification of the appropriate level for tests and full reporting of outcomes
☒	☐	Estimates of effect sizes (e.g. Cohen's *d*, Pearson's *r*), indicating how they were calculated

Our web collection on statistics for biologists contains articles on many of the points above.

Software and code

Policy information about availability of computer code

Data collection	SLS-PXIII DA+ ; ESRF ID23-2 MX-Cube
Data analysis	XDS VERSION Jan 26, 2018 BUILT=20180409; SHELX: Version 2018/3; hkl2map-Version 0.4.e-beta; coot-0.8; phenix-1.13_2998; Phenix-phaser Version:2.8.1, phyre2-v2.0; pymol-2.1.0; Microcal-peaq-itc Version-1.22; Clustal Omega (https://www.ebi.ac.uk/Tools/msa/clustalo/)

For manuscripts utilizing custom algorithms or software that are central to the research but not yet described in published literature, software must be made available to editors and reviewers. We strongly encourage code deposition in a community repository (e.g. GitHub). See the Nature Research guidelines for submitting code & software for further information.

Data

Policy information about availability of data

All manuscripts must include a data availability statement. This statement should provide the following information, where applicable:

- Accession codes, unique identifiers, or web links for publicly available datasets
- A list of figures that have associated raw data
- A description of any restrictions on data availability

PDB ID code of the structures deposited in the Protein Data Bank is reported in the manuscript: PDB-ID 6ZSY, 6ZSZ, and 6ZT0.

Field-specific reporting

Please select the one below that is the best fit for your research. If you are not sure, read the appropriate sections before making your selection.

☒ Life sciences ☐ Behavioural & social sciences ☐ Ecological, evolutionary & environmental sciences

For a reference copy of the document with all sections, see nature.com/documents/nr-reporting-summary-flat.pdf

Life sciences study design

All studies must disclose on these points even when the disclosure is negative.

Sample size	For immunofluorescence, stainings of discs from 20 animals was observed for each experiment. Given the reproducibility of the results, this sample size was considered sufficient to obtain significative results.
Data exclusions	No data were excluded from the analyses.
Replication	All immunofluorescence and western blotting experiments have been repeated 3 times and the most representative acquisition is shown. SLS experiments in Figure 1D were confirmed by successful experiments replicated at least twice. ITC experiments of Figure 1E were confirmed by successful experiments replicated at least twice. Pull-down and co-IP experiments of Figure 3H, 3L and 3M were repeated at least 3 times.
Randomization	Randomization was not applicable as we do not report population studies.
Blinding	Blinding was not applicable as we do not report population studies.

Reporting for specific materials, systems and methods

We require information from authors about some types of materials, experimental systems and methods used in many studies. Here, indicate whether each material, system or method listed is relevant to your study. If you are not sure if a list item applies to your research, read the appropriate section before selecting a response.

Materials & experimental systems

n/a	Involved in the study	
☐	☒	Antibodies
☒	☐	Eukaryotic cell lines
☒	☐	Palaeontology and archaeology
☐	☒	Animals and other organisms
☒	☐	Human research participants
☒	☐	Clinical data
☒	☐	Dual use research of concern

Methods

n/a	Involved in the study	
☒	☐	ChIP-seq
☒	☐	Flow cytometry
☒	☐	MRI-based neuroimaging

Antibodies

Antibodies used	The following primary antibodies were used for immunofluorescence in this study: guinea pig anti-Grnd (1/500) described in (Andersen et al., 2015), mouse anti-Wgn from DSHB 1/50 (#4D4; https://dshb.biology.uiowa.edu/4D4), rabbit anti-cleaved caspase-3 (Asp 175) from Cell Signaling Technology 1/500 (#9661; https://www.cellsignal.com/products/primary-antibodies/cleaved-caspase-3-asp175-antibody/9661), and mouse anti-Wgn (1/100) described in (Andersen et al., 2015).<br>The following secondary antibodies were used : Cy3 conjugated donkey anti-rabbit, Cy3 conjugated donkey anti-mouse and Cy3 conjugated mouse anti-guinea pig, and Cy5 conjugated donkey anti-mouse from Jackson ImmunoResearch; goat anti-mouse Alexa Fluor 488 from Invitrogen).<br>The following primary antibodies were used for western blotting: mouse anti-Flag from Sigma (#F3165; https://www.sigmaaldrich.com/catalog/product/sigma/f3165?lang=en&region=DK), rat anti-HA from Roche (#3F10; https://www.fishersci.com/shop/products/anti-ha-high-affinity-50-ug/501003325).
Validation	All used antibodies have previously been validated and published, or are available commercially.

Animals and other organisms

Policy information about studies involving animals; ARRIVE guidelines recommended for reporting animal research

Laboratory animals	drosophila melanogaster<br>Transgenic flies : the w1118; +; rn-Gal4, UAS-Egr, tubGal80ts/TM6B line was provided by I. Hariharan, Dept of Molecular & Cell Biology, University of California, Berkeley, USA. The UAS-egr-venus line was a gift from Marcos Vidal, Beatson Institute for Cancer

Research, Glasgow, UK. The en-Gal4, UAS GFP/Cyo line was provided by Bruce A. Edgar, Huntsman Cancer Institute, Salt Lake City, USA. The rn-Gal4 (BL7405) line was provided by the Bloomington Drosophila Stock Center. The following RNAi lines were from the collections of the Vienna Drosophila RNAi Center (VDRC): tak1 RNAi (KK101357) and wgn RNAi (GD9152).
grndWT, grndKO, grndH66A, grndF46A , and grndH66A,N67A mutant flies have been generated for this paper and data is provided.
Immunofluorescence experiments are done on wing imaginal discs from third instar (5 day old) male and female larvae .

Wild animals	No wild animals were used in this study.
Field-collected samples	No field-collected samples were used in this study.
Ethics oversight	This study does not require ethical approval.

Note that full information on the approval of the study protocol must also be provided in the manuscript.
